# Changes in the content of thiol compounds and the activity of glutathione s-transferase in maize seedlings in response to a rose-grass aphid infestation

**DOI:** 10.1371/journal.pone.0221160

**Published:** 2019-08-14

**Authors:** Iwona Łukasik, Aleksandra Wołoch, Hubert Sytykiewicz, Iwona Sprawka, Sylwia Goławska

**Affiliations:** Siedlce University of Natural Sciences and Humanities, Department of Biochemistry and Molecular Biology, Siedlce, Poland; Universidade de Sao Paulo Instituto de Biociencias, BRAZIL

## Abstract

The rose-grass aphid (*Methopolophium dirhodum* Walk.) is a major pest of maize (*Zea mays* L.), but little is known about the biochemical interactions between *M*. *dirhodum* and its host plant. Thiol compounds and glutathione S-transferase (GST) play a crucial role in the defense responses of maize to biotic stress factors, including aphids. The purpose of this research was to evaluate the impact of *M*. *dirhodum* herbivory on the total thiol (TT), protein bound thiol (PT), reduced glutathione (GSH) and oxidized glutathione (GSSG) contents as well as the activity of GST in three varieties of *Z*. *mays* (Złota Karłowa, Ambrozja and Płomyk), that were classified as aphid-susceptible, aphid-relatively resistant and aphid-resistant, respectively. The earliest and strongest aphid-triggered alterations in the levels of TT, PT and GSH, and the greatest induction of GST activity, were recorded in the resistant Płomyk seedlings in relation to the relatively resistant Ambrozja and the susceptible Złota Karłowa.

## Introduction

Maize (*Zea mays* L.) is one of the most important crops worldwide and its economic importance is growing [1]. Maize is a substantial source of raw materials for the pulp and paper industries as well as for the fermentation processes in biogas and bioethanol production [2–4]. *Z*. *mays* serves as a model organism in experimental biology, especially in studies related to plant-insect interactions and resistance mechanisms [2–3]. Among numerous insects attacking *Z*. *mays*, aphids are one of the major pests responsible for damage to maize [5–6]. Four species of aphids infest maize plants in Poland: the rose-grass aphid *Metopolophium dirhodum* (Walk.), the bird cherry-oat aphid *Rhopalosiphum padi* (L.), the corn leaf aphid *Rhopalosiphum maidis* (F.) and the grain aphid *Sitobion avenae* (F.) [6–9]. The injection of aphid saliva can be very toxic, leading to chlorosis, deformation of organs, disturbance of water transport and depletion of chlorophyll contents [10]. Aphids are also associated with the transmission of a wide spectrum of plant viruses, including barley yellow dwarf virus (BYDV), beet western yellows virus (BWYV), maize dwarf mosaic virus (MDMV), soybean dwarf virus (SbDV) and sugarcane mosaic virus (SCMV) [11–15].

Aphid feeding may trigger multiple signalling pathways in plants, which are mediated by several molecules, including jasmonic acid, salicylic acid, ethylene, abscisic acid, gibberellic acid, nitric oxide and reactive oxygen species (ROS) [16–19]. The rapid increase in ROS generation, called an oxidative burst, is one of the earliest plant responses to aphid infestation [20–22]. Excess ROS is harmful to plants, as it can cause lipid peroxidation, protein oxidation, damage to DNA and activation of programmed cell death (PCD) [23–24]. Whether ROS play a damaging or signalling role depends on the ROS generation and elimination by the plant’s defense system, composed of antioxidant enzymes, such as superoxide dismutase (SOD), catalase (CAT), glutathione peroxidase (GPX) and ascorbate peroxidase (APX), and non-enzymatic antioxidants, such as ascorbic acid (ASA), tocopherols and glutathione (GSH) [23], [25]. Thiols and glutathione (GSH; γ-L-glutamyl-L-cysteinylglycine) are two of the crucial metabolites that act as detoxicants and antioxidants [26–27]. Equilibrium between GSH and its oxidised form (glutathione disulfide–GSSG) is a fundamental requirement for maintaining a cellular redox state [28]. GSH plays an important role in many biological processes, including cell growth, signal transduction, synthesis of proteins and nucleic acids as well as detoxification of a wide spectrum of xenobiotics [23], [26], [29–31]. GSH is a disulfide reductant that protects protein thiol groups, reacts directly with hydrogen peroxide (H_2_O_2_) and hydroxyl radical (·OH), and acts as the substrate for glutathione-dependent enzymes, such as GPX, dehydroascorbic acid reductase (DHAR) and glutathione S-transferase (GST) [32–33]. Cytosolic GSTs are a family of multifunctional enzymes that participate in conjugation and sequestration of xenobiotics, transport of flavonoids, programmed cell death and signalling through flavonoids [34]. Some isoforms of GST show dual activity and can also act as glutathione peroxidases (GSTpx), removing organic hydroperoxides [35–36].

There are numerous studies concerning the role of thiol compounds and GST in plants under various stresses (herbicides, salinity, heavy metals, fungal and viral infection, herbivores [19], [27], [37–42]. In the last years, the impact of two cereal aphid species (*R*. *padi* L. and *S*. *avenae* F.) on the expression patterns of genes related to GSH and GST in *Z*. *mays* seedlings has been intensively studied [19], [27], [42]. However, there is a lack of published data concerning the effect of *M*. *dirhodum* infestation on glutathione metabolism in maize. *M*. *dirhodum* is a host-alternating aphid, whose primary hosts are roses (*Rosa* L.) and secondary hosts are grass species, mainly cereals [43]. Studies conducted by Bereś [6] proved that the most abundant aphid species occurring on maize in Poland are *R*. *padi* and *M*. *dirhodum*. Moreover, Strażyński [9] noted that *M*. *dirhodum* was the predominant species on maize cultivars in the Wielkopolska region. Therefore, the aim of the present study was to determine the changes in levels/activities of total thiols (TT), protein thiols (PT), GSH, GSSG and GST in seedlings of three maize cultivars, differing in aphid resistance (Złota Karłowa, Ambrozja and Płomyk) after a *M*. *dirhodum* infestation, in order to gain a better insight into the thiol metabolism in the examined host plants.

## Methods

### Aphids

Experiments were conducted using wingless females (*apterae*) of the rose-grass aphid *M*. *dirhodum*. The aphids were reared on the seedlings of wheat (*Triticum aestivum* L.) cv. Tonacja for a year in an environmental chamber (21°C, L16:D8 photoperiod, 70% relative humidity).

### Plants

The seeds of three *Z*. *mays* varieties (Ambrozja, Złota Karłowa and Płomyk) were acquired from local commercial grain suppliers: Reheza (Moszna, Poland) and PNOS S.A. (Ożarów Mazowiecki, Poland). Plants were cultivated in a climate chamber (21°C, L16:D8 photoperiod, 70% relative humidity). The seedlings were grown in plastic pots (10 ×10 cm, one seedling per pot) filled with medium nutrient fine structure compost with sand. According to Sytykiewicz et al [10], the Złota Karłowa, Ambrozja and Płomyk maize varieties are classified as aphid-susceptible, aphid-relatively resistant and aphid resistant, respectively.

### Infestation procedure

Leaves of 14-day-old maize seedlings were colonized with 30 or 60 adult wingless *M*. *dirhodum* females per plant. The control groups of seedlings were not infested with insects. Maize plants infested with aphids and the non-infested (control) plants were isolated in gauze-covered plastic cylinders. The quantified parameters (i.e. TT, PT, GSH, GSSG and GST) in maize plants were estimated after 24, 48, 72 and 96 h of the continuous aphid infestation.

### Quantification of TT, non-protein thiols (NPT) and PT

TT and PT contents were determined according to Sedlak and Lindsay [44]. Fresh *Z*. *mays* seedling leaves weighing 500 mg were homogenized in 10 ml of 0.2M Tris-HCl (pH 7.4) and centrifuged at 10 000 *x g* for 20 min at 4°C. Supernatant was used to assay TT and NPT. To determine the TT, 0.5 ml of supernatant was mixed with 1.5 ml of 0.2mM Tris-HCl (pH 8.2), 0.1 ml of 0.01 M DTNB and 7.9 ml of absolute methanol. The yellow colour that developed was measured after 15 min at 415 nm against a blank vial containing 0.5 ml distilled water instead of supernatant. Total sulfhydryl groups were calculated based on an extinction coefficient of 13,600 and expressed as μmol per g fresh weight.

To determine of NPT content, 5 ml of supernatant was mixed with 4 ml of distilled water and 1 ml of 50% TCA. After 15 min. the mixture was centrifuged at 10 000 *x g* for 15 min. In 2 ml of deproteinized supernatant, NPT concentration was measured in a manner similar to that for TT [44].

PT were calculated by subtracting the NPT content from total thiol content.

### GSH and GSSG assay

The contents of GSH and GSSG were determined as described by Griffith [45] based on the oxidation of GSH by DTNB [5,5’-dithiobis-(2-nitrobenzoic acid)] to form GSSG and TTNB (5-thio-2-nitrobenzen). GSSG was reduced to GSH by the glutathione reductase and NADPH. Briefly, 500 mg of plant material was homogenized in 2.5 ml of 2.5% TCA and centrifuged at 10 000 *x g* for 15 min at 4°C. A 0.3 ml of the supernatant was used to assay total glutathione (GSH + GSSG). Another 0.3 ml was pretreated with 6 μl 2-vinylpyridine for 60 min at 20°C to mask GSH by derivatization. 0.1 ml of both types of samples were mixed with 0.7 ml of 0.3 mM NADPH, 0.1 ml of 6 mM DTNB and 0.1 ml of GR (50 units/ml). The absorbance at 412 nm was recorded after 5 min at room temperature. The total glutathione (GSH + GSSG) and GSSG contents were calculated using a standard curve and was expressed as μmol per g fresh weight. The GSH content was calculated from the difference between the total glutathione and GSSG.

### GST assay

GST activity was measured using 1-chloro-2,4-dinitrobenzene (CDNB), as per Leszczyński and Dixon [46]. 500 mg of plant material was homogenized in 5 ml of 0.2M Na-phosphate buffer pH 7.8 and centrifuged it at 10 000 *x g* for 15 min at 4°C; the obtained supernatant was used to assay GST. The reaction mixture contained 1 ml of crude supernatant and 0.05 ml of 50 mM GSH. After incubation for 5 min at 25°C, 0.025 ml of 40 mM CDNB was added into the mixture and absorbance at 340 nm was recorded for 5 min. GST activity was expressed as nmol CDNB conjugated/min/g fresh weight, using extinction coefficient of 9.6 mM^-1^ x cm^-1^ for S-(2,4-dinitrophenyl)glutathione.

### Statistical analysis

All data are reported as means ± SD and *n* = 4, where each replication represents one independent plant homogenate. Differences in the content of TT, PT, GSH, GSSG and activity of GST between the aphid-infested seedlings of each variety and the relevant control plants were assessed by an analysis of variance (ANOVA), followed by a post hoc Fisher’s LSD test. All analyses were done with Statistica for Windows version 10.0 (Statsoft 2012).

## Results

### Effects of the rose-grass aphid infestation on the TT content in *Z*. *mays* seedlings

The experiment revealed that the exposure of the seedlings of three maize cultivars (Ambrozja, Złota Karłowa and Płomyk) to *M*. *dirhodum* caused significant fluctuations in TT content in comparison to control non-infested plants (Table 1). Insect feeding for 24 h on Ambrozja seedlings resulted in a clear increase in the level of TT, dependent on the number of aphids per plant. Lower insect density (30 insects per seedling) caused larger changes in the level of TT in the tested plants. Forty-eight hours after colonization, a lower abundance of aphids (30 per plant) did not affect the TT content in Ambrozja cultivar leaves in relation to the uninfested control, but a higher density of aphids (60 per plant) evoked depletion of the TT level. Over the next two time periods (72 and 96 h) of the rose-grass aphid infestation, a gradual decrease in the sulfhydryl compound content in Ambrozja cultivar leaves was noted. At 72 h post-infestation, the extent of depletion was independent of the aphid density, but after 96 h of insect feeding, a greater decrease in TT content was noted for plants stressed by a higher number of aphids (60 per seeding).

**Table 1:** Changes in the total thiol content (μmol/g fresh weight) in maize seedlings after the rose-grass aphid infestation.

Cultivar	Control (non-infested)	Feeding time(h)							
		24		48		72		96	
		30 aphids	60 aphids	30 aphids	60 aphids	30 aphids	60 aphids	30 aphids	60 aphids
Ambrozja	8.13±0.68b	9.86±0.24a	7.20±0.18c	8.31±0.35b	6.52±0.28cd	7.06±0.22c	6.44±0.31cd	6.28±0.30d	4.62±0.19e
Złota Karłowa	6.45 ±1.36cd	7.12±0.46bc	7.43±0.33b	7.43±0.29b	6.95±0.40bc	8.20±0.22a	6.74±0.36c	7.26±0.40bc	6.03±0.33d
Płomyk	9.3±0.36a	9.40±0.40a	7.99±0.23bc	7.40±0.18d	7.69±0.31cd	8.28±0.34b	8.28±0.31b	9.20±0.23a	4.41±0.37e

Values are means ± standard deviation (SD) of four independent plant homogenates; different letters in rows denote significant differences according to Fisher’s LSD test (p<0.001)

In the case of the Złota Karłowa cultivar, the initial periods of *M*. *dirhodum* colonization (24 and 48 h) were associated with an increase in TT content in comparison to control maize seedlings. At these time points, the intensity of changes was comparable and independent of aphid density. Prolonged aphid feeding (72 h) evoked a further elevation of TT concentrations only for the lower abundance of aphids (30 per plant). The maize seedlings infested with 60 aphids had a TT level comparable to control plants, but lower than during the initial period of experiment (24 h). Continued aphid feeding on Złota Karłowa seedlings (96 h) at a lower density (30 per plant) resulted in an increase in TT content, whereas a higher abundance of aphids (60 per plant) did not evoke any alterations compared to the non-infested control plants (Table 1).

The initial feeding of aphids (24 h) on Płomyk cultivar seedlings did not alter TT content at a lower density of insects, whereas plants infested by a higher number of females exhibited a decreased TT level compared to the control, non-stressed maize seedlings. Over the next studied time points (48 and 72 h), we observed a decrease in TT concentration in infested plants, with the level of depletion being independent of aphid density. After 96 h of infestation, the lower abundance of aphids did not alter the TT content in maize plants, but a higher density of aphids reduced TT concentration by about 50% relative to control seedlings (Table 1).

Among tested maize cultivars, Płomyk plants responded with the greatest TT content depletion (53% decline at 96 h post infestation with 60 aphids) while the lowest reduction of total -SH was noted for Złota Karłowa (only 7% relative to non-infested plants after 96 h with 60 aphids per plant) (Table 1).

### Effects of the rose-grass aphid infestation on the PT content in *Z*. *mays* seedlings

The conducted experiment showed that the PT content of Ambrozja cultivar tissues decreased after colonization by *M*. *dirhodum*, with the exception of a lower density of aphids (30 per plant) which caused an increase (24 h post-infestation) or did not alter PT content (48 h post-infestation). PT levels were approximately equal after two periods of the colonization (48 and 72 h) for both studied densities of insects. Maximal depletion of PT was observed after 96 h of *M*. *dirhodum* feeding. The depletion of protein sulfhydryls was dependent on the abundance of aphids on the tested maize seedlings. Ambrozja plants infested with 60 *M*. *dirhodum* females were characterised by a lower level of PT than seedlings treated with 30 insects per plant(Table 2).

**Table 2:** Changes in the protein thiol content(μmol/g fresh weight) in maize seedlings after the rose-grass aphid infestation.

Cultivar	Control (non-infested)	Feeding time (h)							
		24		48		72		96	
		30 aphids	60 aphids	30 aphids	60 aphids	30 aphids	60 aphids	30 aphids	60 aphids
Ambrozja	6.35±0.43b	7.37±0.41a	5.71±0.22c	6.20±0.26bc	4.70±0.32d	5.46±0.31c	4.51±0.24d	4.88±0.47d	2.60±0.26e
Złota Karłowa	5.95±0.33a	6.00±0.51a	5.79±0.27ab	5.98±0.51a	5.35±0.31bc	5.95±0.37a	5.06±0.36c	5.12±0.47c	4.1±0.35d
Płomyk	6.47±0.31ab	7.12±0.37a	5.24±0.32de	4.65±0.39e	5.05±0.54de	5.5±c0.30d	5.37±1.01cde	6.08±0.54bc	2.07±0.35f

Values are means ± standard deviation (SD) of four independent plant homogenates; different letters in rows denote significant differences according to Fisher’s LSD test (p<0.001)

Different results were obtained for Złota Karłowa, where the concentration of PT was remained unaffected until 72 h of infestation when evaluated at a lower number of aphids. The colonization of Złota Karłowa plants by a higher number of insects (60 per seedling) did not evoke any alterations after 24 h, but significantly limited the PT level over the next studied periods (48 and 72 h). After 96 h, the content of protein–SH groups was limited in the maize tissues for both studied densities of *M*. *dirhodum*, although a stronger depletion was noted in the seedlings treated with a higher number of aphids (Table 2).

Insect feeding on Płomyk seedlings resulted in a decline in PT content, with the exception of two aphid treatments (30 aphids per plant at 24h and 96h) when there were no changes in the concentration of PT in comparison to control plants. The level of protein sulfhydryls depletion was comparable for two periods of infestation (24 h and 48 h), but the strongest reduction in PT content was noted at 96 h post-infestation with a higher number of aphids (Table 2).

The strongest decline in PT was observed in Płomyk seedlings (60 aphids per plant at 96 h) and the lowest was noted in Złota Karłowa plants (10%-31% decline in relation to the control) (Table 2).

### Effects of the rose-grass aphid infestation on the GSH content in *Z*. *mays* seedlings

The GSH content in Ambrozja plants decreased after colonization by *M*. *dirhodum* and the intensity of alterations depended on the aphid density over the two initial periods of the experiment (24 and 48 h) (Table 3). At these time points, Ambrozja plants colonized by 60 aphids were characterized by a lower GSH content than seedlings stressed by 30 insects per plant. The extent of GSH depletion in Ambrozja cultivar was independent of the duration of feeding.

**Table 3:** Changes in the reduced glutathione content (μmol/g fresh weight) in maize seedlings after the rose-grass aphid infestation.

Cultivar	Control (non-infested)	Feeding time (h)							
		24		48		72		96	
		30 aphids	60 aphids	30 aphids	60 aphids	30 aphids	60 aphids	30 aphids	60 aphids
Ambrozja	0.33±0.04a	0.24±0.04b	0.15±0.05c	0.22±0.04b	0.16±0.04c	0.22±0.03b	0.20±0.03bc	0.24±0.02b	0.20±0.02bc
Złota Karłowa	0.45±0.05a	0.36±0.04b	0.28±0.06bc	0.32±0.07b	0.27±0.05bc	0.35±0.05b	0.30±0.06bc	0.30±0.06bc	0.22±0.02c
Płomyk	0.25±0.03a	0.11±0.02c	0.07±0.02c	0.10±0.02c	0.10±0.02c	0.16±0.02b	0.15±0.03b	0.18±0.03b	0.17±0.03b

Values are means ± standard deviation (SD) of four independent plant homogenates; different letters in rows denote significant differences according to Fisher’s LSD test (p<0.001)

A similar tendency was observed in Złota Karłowa plant tissues, but no dependence between GSH depletion and aphid density was noted at all studied periods of experiments (Table 3). Different results were obtained in the case of the Płomyk cultivar, where the GSH level depleted nearly two-fold after 24 and 48h of infestation for both studied aphid densities (30 and 60 per plant). Prolonged aphid feeding (72 and 96h)evoked an increase in the GSH content in relation to earlier periods of infestation, but the GSH level in stressed Płomyk seedlings remained lower in comparison to non-infested control plants (Table 3).

Among the tested cultivars of maize, Płomyk seedlings were characterized by the highest depletion in GSH content after 24 and 48 h post infestation, while Złota Karłowa plants exhibited the largest GSH loss at the end of the experiment (96 h) (Table 3).

### Effects of the rose-grass aphid infestation on the GSSG content and the GSH/GSSG ratio in *Z*. *mays* seedlings

It has been revealed that the GSSG amount remained stable in seedlings of two studied cultivars (Ambrozja and Złota Karłowa) infested with 30 aphids for 24 and 48h. Prolonged feeding (72–96 h) at lower aphids’ density was linked to increase in GSSG level in foliar tissues of Ambrozja and Złota Karłowa cultivars. Furthermore, the lower abundance of aphids did not evoke significant alternations in the GSSG content in Płomyk seedlings for up to 96 h. The colonization of Ambrozja and Złota Karłowa plants by a higher number of insects (60 per seedling) resulted in the elevations in GSSG content at all studied periods of the infestation (24, 48, 72 and 96 h). The intensity of alterations in Ambrozja seedlings was independent on duration of feeding, whereas Złota Karłowa plants stressed by 60 insects had the highest GSSG level at 96 h post-infestation. Different results were obtained for Płomyk, where initial periods of aphid feeding (24 and 48 h) at higher density evoked significant increase in GSSG content, but prolonged feeding (72 and 96h) did not affect any changes in infested plants in comparison with the control. At 24 and 48 h post-infestation, the highest elevations in GSSG amount were observed in Płomyk seedlings, whereas the prolongation of aphid feeding (96 h) evoked the strongest increase in GSSG content in Złota Karłowa plants (Table 4).

**Table 4:** Changes in the oxidized glutathione content (μmol/g fresh weight) in maize seedlings after the rose-grass aphid infestation.

Cultivar	Control (non-infested)	Feeding time (h)							
		24		48		72		96	
		30 aphids	60 aphids	30 aphids	60 aphids	30 aphids 6	60 aphids	30 aphids	60 aphids
Ambrozja	0.10±0.01c	0.12±0.01bc	0.16±0.01a	0.12±0.0 bc	0.13±0.01ab	0.13±0.01b	0.14±0.02ab	0.12±0.01b	0.13±0.01ab
Złota Karłowa	0.15±0.01e	0.17±0.01e	0.20±0.01bc	0.16±0.01e	0.20±0.01cd	0.19±0.01d	0.21±0.02bc	0.22±0.01b	0.25±0.02a
Płomyk	0.04±0.01b	0.06±0.01ab	0.07±0.01a	0.06±0.01ab	0.07±0.02a	0.05±0.01ab	0.06±0.01ab	0.04±0.01b	0.05±0.01ab

Values are means ± standard deviation (SD) of four independent plant homogenates; different letters in rows denote significant differences according to Fisher’s LSD test (p<0.001)

The seedlings of three investigated maize cultivars (Ambrozja, Złota Karłowa and Płomyk) infested with *M*. *dirhodum* characterized with significant depletion in the GSH/GSSG ratio in relation to control plants. The higher decrements in the level of the studied parameter in the seedlings colonized by 60 aphids were observed. Additionally, Płomyk cultivar had the greatest depletion in the GSH/GSSG ratio at the initial periods of infestation (24 and 48 h), whereas the long-term aphids’ feeding led to the largest decrement in the estimated parameter in Złota Karłowa plants (Table 5).

**Table 5:** Changes in the GSH/GSSG ratio in maize seedlings after the rose-grass aphid infestation.

Cultivar	Control (non-infested)	Feeding time (h)							
		24		48		72		96	
		30 aphids	60 aphids	30 aphids	60 aphids	30 aphids 6	60 aphids	30 aphids	60 aphids
Ambrozja	3.36±0.42a	2.07±0.32b	0.98±0.27e	1.85±0.09bc	1.21±0.21de	1.76±0.08bc	1.54±0.41cd	1.98±0.16b	1.52±0.11cd
Złota Karłowa	2.97 ±0.28a	2.17±0.35b	1.36±0.30cd	1.99±0.51b	1.40±0.35cd	1.88±0.17bc	1.43±0.31c	1.39±0.33cd	0.89±0.09d
Płomyk	6.17±0.91a	2.02±0.38c	1.03±0.05d	1.62±0.11cd	1.58±0.40cd	3.72±0.95b	2.51±0.24c	4.61±0.67b	3.63±0.75b

Values are means ± standard deviation (SD) of four independent plant homogenates; different letters in rows denote significant differences according to Fisher’s LSD test (p<0.001)

### Effects of the rose-grass aphid infestation on the GST activity in *Z*. *mays* seedlings

The colonization of Ambrozja cultivar by a lower number of aphids (30 per plant) did not influence GST activity over the two initial periods of the infestation (24 and 48 h). The next studied periods (72 and 96 h) were associated with a strong increase in GST activity with the level of induction being comparable at these time points. Infestation by a higher number of insects (60 per seedlings) resulted in the activation of GST at all studied periods of the experiment (24, 48, 72 and 96 h) and the level of induction was independent of the time of aphid feeding. No differences in the activity of GST were noted between the two studied densities of aphids at all time points of the experiment (Table 6).

**Table 6:** Changes in the activity of glutathione transferase (nmol CDNB conjugated/min/g fresh weight) in maize seedlings after the rose-grass aphid infestation.

Cultivar	Control (non-infested)	Feeding time(h)							
		24		48		72		96	
		30 aphids	60 aphids	30 aphids	60 aphids	30 aphids	60 aphids	30 aphids	60 aphids
Ambrozja	15±1.83c	18±3.83bc	20±2.94ab	19±3.56abc	20±3.37ab	21±3.37ab	21±3.92ab	23±3.74a	21±3.27ab
Złota Karłowa	22±2.58d	24±3.65cd	25±3.83cd	24±3.16cd	27±4.08bcd	25±3.74bcd	30±4.55bc	26±3.74b	35±3.16a
Płomyk	16±1.29e	24±2.58d	24±3.16d	25±3.37cd	28±2.94bcd	29±2.94bc	33±2.94a	32±2.71ab	38±2.93a

Values are means ± standard deviation (SD) of four independent plant homogenates; different letters in rows denote significant differences according to Fisher’s LSD test (p<0.001)

Infestation of Złota Karłowa plants with the two aphid densities (30 and 60 per plant) did not alter GST activity after 24 and 48 h of aphid feeding. Changes in the GST activity level at the next analysed period (72 h) was dependent of the aphid density; a lower number of aphids did not affect enzyme activity in comparison to the non-infested control, but a higher abundance of insects (60 per seedling) induced GST activity. After 96 h of infestation, GST activity level was enhanced in Złota Karłowa plants colonized by both densities of aphids (30 and 60 per plant), but a stronger activation was noted for seedlings stressed by more *M*. *dirhodum* females. It was maximal induction of GST activity during infestation of Złota Karłowa seedlings by the rose-grass aphid (Table 6).

The exposure of Płomyk seedlings to *M*. *dirhodum* adults caused an increase in GST activity and the level of induction was comparable after 24 h and 48 h of infestation with both studied densities of aphids. Prolonged aphid feeding resulted in a progressive induction of GST activity, which was dependent on the density of aphids only 48 h post infestation. Similarly to the other cultivars, the maximal enhancement in GST activity in Płomyk seedlings was observed at the end of experiment (96 h post infestation) (Table 6).

The greatest increases in GST activity were noted in Płomyk seedlings, whereas the lowest induction was recorded in Złota Karłowa plants, excluding the last period of experiment, when infestation with 60 aphids caused the least increment in GST activity in Ambrozja seedlings (Table 6).

## Discussion

Thiols function as antioxidants due to their reductive ability and capacity to react with ROS. The redox state of thiols plays an important role in the determination of protein structure, regulation of enzyme activities, oxidative stress control and protection against xenobiotics [47–48]. The biological significance of thiol compounds is related to the activity of the sulfhydryl group involved in antioxidant and detoxification reactions [47]. In protein molecules, the amino acids containing thiol groups and sulphur are the most susceptible sites for ROS action [23], [49]. ROS can react with cysteine residues to form a sulfenic acid or thiyl radical that can subsequently lead to the formation of several products, including disulfides [23]. The loss of protein–SH groups leads to protein misfolding, catalytic inactivation and diminution of antioxidant potential [49]. In plant cells is present a system controlling the dithiol-disulfide interchanges of target proteins which consists of thioredoxin (TRX), peroxiredoxin (PRX) and NADPH-thioredoxin reductase (NTR) [50]. TRXs are oxidoreductases containing two cysteines in the redox active site which catalyse the reduction of disulfide to dithiol in proteins [50, 51]. This reaction led to the TRXs oxidation, thus TRXs are reduced back by NTR [52]. TRXs return PRXs to their reduced forms to maintain their ability to reduce of peroxides [50].

Exposure to a higher number of *M*. *dirhodum* (60 per plant) caused a depletion of TT in tissues of relatively resistant (Ambrozja) and resistant (Płomyk) cultivars of maize. In the case of the susceptible variety (Złota Karłowa), we noted a decrease in the TT content only after 96 h post infestation with higher number of aphids. The strongest depletion of TT was observed in the tissues of the resistant cultivar Płomyk. Many reports have demonstrated changes in the concentration of thiol compounds under abiotic stress factors, but little is known about the role of thiols in host plant responses to biotic stressors of arthropod origin, including sucking-piercing insects. Bhoomika et al. [53] observed no significant alternations in the TT content of rice seedlings exposed to aluminum. Kaur et al. [54] revealed that earthworm supplementation to cadmium-treated soils increased the level of TT in *Brassica juncea* L. plants. Nemat Alla and Hassan [55] stated that isoproturon treatment led to large increases in the TT content of maize plants, but that the thiols were induced only during the first few days and by low doses. Thus, changes in the concentration of TT in plants seem to depend on the type of stress and the intensity of oxidative stress. Some metals or herbicides may induce the synthesis of thiols, whereas biotic stressors (e.g. aphids) can cause the depletion of sulfhydryl compounds.

The results of this study demonstrated that the earlier and more substantial reduction of PT content after *M*. *dirhodum* infestation occurred in seedlings of the resistant variety Płomyk. However, the lower abundance of aphids (30 per plant) at 24 h of infestation did not affect the content of protein-bound sulfhydryl content in Płomyk cultivar leaves. This is opposite to the results obtained by Bhoomika et al. [53], where aluminum treatment caused a consistent decline in PT in Al-sensitive rice cultivar, whereas the content of protein sulfhydryls in the seedlings of Al-tolerant cultivar remained unchanged. The authors suggest that the induction of ROS production and the oxidative stress induced by aluminum was greater in the Al-sensitive cultivar seedlings than in those of the Al-tolerant cultivar. Aly and Mohamed [32] noted that the level of protein-bound thiols in *Z*. *mays* significantly increased by increasing the copper levels in the growth media. Similar results were obtained by Kaur et al. [54], where earthworm supplementation to cadmium-treated soils increased the content of protein sulfhydryls in *B*. *juncea* L. plants. However, a depletion of protein bound–SH groups was seen in many plants subjected to various types of abiotic stress. The significant reduction of PT (18.9%) was noted in radish (*Raphanus sativus* L.) seedlings exposed to zinc stress [56]. Salt stress resulted in a depletion of protein sulfhydryl groups in embryogenic suspension cultures of *Dactylis glomerata* L. [57]. Gietler et al. [58] revealed that dehydration resulted in a significant decrease in the protein-bound thiol content in *Triticum aestivum* L. seedlings; the concentration of protein sulfhydryls was more severely reduced in the sensitive seedlings than in the tolerant ones.

GSH is a major non-enzymatic antioxidant, whose function is mediated by the cysteinyl thiol group that, upon oxidation, forms GSSG [33]. In the current work the infestation of *Z*. *mays* plants by a higher number of aphids (60 per plant) caused a decrease in GSH in all studied cultivars, but the resistant Płomyk cultivar was characterized by the strongest reduction in GSH level at initial periods of aphid infestation. Together with GSH depletion, we observed explicit increase in GSSG content and decrement in the GSH/GSSG ratio, what indicates the induction of oxidative stress in maize plants stressed *by M*. *dirhodum*. Kar et al. [41] stated that leaves of *Terminalia arjuna* Arjun infested with sap-sucking *Trioza fletcheri* Crawford responded with a significant depletion in GSH levels, in relation to the non-infested control. Furthermore, the GSH content substantially decreased in the leaves of cabbage (*Brassica oleracea* L.) infested with the cabbage aphid *Brevicoryne brassicae* L., compared to healthy, uninfested cabbage leaves [59]. Sytykiewicz [27] demonstrated a gradual depletion in the GSH content in tissues of two tested maize genotypes (relatively resistant Ambrozja and susceptible Tasty Sweet) 8, 12 and 24 hours post-infestation with *S*. *avenae* and *R*. *padi*. The aphid-infested seedlings of the more resistant maize genotype (Ambrozja cultivar) reacted with a greater progressive depletion in GSH content. Moreover, a gradual increase in the GSSG level in Ambrozja and Tasty Sweet from 2 to 24 h post infestation was revealed, but prolonged aphid feeding was linked to significant GSSG elevation in Tasty Sweet seedlings and minor alterations in Ambrozja plants [27]. Additionally, the aphid-infested leaves of more resistant maize genotype (Ambrozja) responded an earlier and higher decrement in the GSH/GSSG ratio in relation to the susceptible variety (Tasty Sweet) [27]. This tendency was confirmed in our study, where the seedlings of resistant cultivar (Płomyk) stressed by *M*. *dirhodum* females, exhibited the lowest the GSH/GSSG ratio over the two initial periods of the experiment. On the contrary, the total glutathione pool of potatoes was enhanced after 48 h of infestation of potato by the peach-potato aphid *Myzus persicae* Sulzer, while the GSH/GSSG ratio remained unchanged [60]. Similarly, Liu et al. [61] demonstrated that the levels of GSH and GSSG increased at the feeding sites in resistant wheat seedlings after Hessian fly (*Mayetiola destructor* Say) infestation, but the GSH/GSSG ratio in infested plants was unaltered in comparison with non-infested (control) seedlings. According to these authors, the level of total glutathione and the GSH/GSSG ratio did not change in infested susceptible plants. Many reports have demonstrated a decrease/increase in GSH concentrations under abiotic stress. Hou et al. [62] evidenced no significant differences in GSH contents between shoots and roots of maize seedlings under mercury stress, but under vanadium-mercury combined stress the GSH content decreased with increasing Hg concentration. Upon salinity or osmotic treatment, the transcripts of genes related to the GSH biosynthesis were significantly increased in transgenic wild type of *Arabidopsis thaliana* L. [63]. During heat stress, GSH content in maize seedlings decreased, but NAHS-treated plants maintained higher GSH level than non-treated controls. Additionally, GSSG content rose up with duration of heat stress, but NAHS-treated maize plants maintained lower GSSG level in comparison to non-treated seedlings [64]. Cadmium remarkably increased the content of total GSH and reduced GSH in maize seedlings in comparison to control plants [65]. GSH level in the shoot and roots of *Z*. *mays* was elevated by salt stress, with higher GSH content in saline/proline-treated seedlings compared with saline/water-treated plants [66].

A reduced GSH level in mitochondria favours accumulation of ROS, which may induce programmed cell death events [31]. In *Nicotiana tabacum* L. plants infected with an incompatible strain of tobacco mosaic virus (TMV) a decrease in GSH content in mitochondria was accompanied by the development of necrotic lesions [67]. In our study, the highest depletion in TT, PT and GSH contents were noted in the resistant Płomyk cultivar and the lowest were noted in the susceptible variety Złota Karłowa. The results of an earlier study demonstrated a more marked elevation in superoxide anion radicals (O_2_ˉ) in the tissues of Ambrozja seedlings (relatively resistant cultivar) in comparison to those of Tasty Sweet (susceptible cultivar) [19]. Additionally, Ambrozja plants infested with *S*. *avenae* and *R*. *padi* showed a more significant decrease in total antioxidant capacity towards DPPH (1,1-diphenyl-2-picnylhydrazyl) radicals, in relation to the susceptible Tasty Sweet cultivar [68]. It seems likely that the stronger depletion of thiol compounds in resistant varieties of *Z*. *mays* markedly depressed the total antioxidant capacity and favored the accumulation of O_2_ˉ in infested maize seedlings.

GSH may directly react with ROS and may be utilised as substrate for reactions catalysed by GST or by GPX. Some GST isoforms exhibit peroxidative activity associated with a reduction of lipid peroxides and the removal of lipid peroxidation products [69]. The performed analysis indicated that *M*. *dirhodum* infestation elevated GST activity in maize seedlings. The highest and earliest inductions of the enzyme were noted in aphid-stressed Płomyk plants. This is in line with results obtained by Sytykiewicz [27], where earlier and more substantial enhancement of GST activity was revealed in Ambrozja (relatively resistant) plants infested with cereal aphids in comparison to Tasty Sweet (susceptible) plants. According to this author, maximal increase in enzyme activity occurred after 24 h post-infestation for both studied maize genotypes. Furthermore, the expression of *gst9*, *gst11*, *gst16*, *gst31* and *gst38* genes was upregulated in maize seedlings in response to stress caused by *S*. *avenae* and *R*. *padi* [27]. Ambrozja plants were characterised by more profound increments in the levels of *gst* transcript in comparison to the Tasty Sweet genotype [19]. A similar pattern was observed by Botha et al. [70] for *T*. *aestivum* after infestation with *Diuraphis noxia* Mordvillko, where the activity of GST was significantly higher in the resistant near-isogenic line in relation to the susceptible and tolerant lines. Similarly to the results of the above-mentioned studies, Moran et al. [71] proved that the feeding of *M*. *persicae* on *Arabidopsis thaliana* L. resulted in nearly three-fold and five-fold elevations in the expression of *gst1* and *gst11* genes, respectively. Stotz et al.[72] elucidated that the diamondback month (*Plutella xylostella* L.) feeding on the rosette leaves of *A*. *thaliana* wild-type led to significant alternations in the expression of *gst2* and *gst6* genes compared to the non-infested control. GST activity was found to be higher in roots of maize colonized by *Fusarium verticilloides* Saccardo as compared to non-colonized plants [73]. The leaves of rice late-infested with mite *Schizotetranychus oryzae* Rossi de Simons exhibited higher GST activity than control leaves [74]. Alternations in GST activity have been demonstrated under the influence of abiotic stress factors. Sytykiewicz [38] revealed that four-days of juglone treatment stimulated the transcriptional activity of *GstI* in maize seedlings compared to the control, but after longer exposure (6 and 8 days) the gene expression responses were lower in relation to non-stressed plants. In contrast, isoproturon significantly reduced GST isoform activities (GST_CDNB_, GST_ALA_ and GST_MET_), but had no effect on GST_ATR_ in maize plants [55]. It is proposed that the induction of GST activity is involved in limiting the cell death events resulting from the elicitation of the hypersensitive type of resistance to pathogenic infections [67], [75–78]. GST may suppress necrosis by detoxifying lipid hydroperoxides produced by the peroxidation of membranes [79].

In conclusion, our experimental results indicate, that the defense mechanisms of maize plants against *M*. *dirhodum* attacks are based on the metabolism of thiol compounds, which is evidenced by alterations in the level of total thiols, protein-bound thiols and GSH, as well as by the activity of GST. The fluctuations in thiol content and GST activity were dependent on the maize variety, the number of aphids and the duration of *M*. *dirhodum* feeding. The strongest modulation of the antioxidant system related to sulfhydryl compounds was noted in the resistant variety, Płomyk, which highlights the role of thiols in the resistance of maize genotypes to aphids.
